# Using Large Language Models for In Silico Development and Simulation of a Patient-Reported Outcome Questionnaire for Cataract Surgery with Various Intraocular Lenses: A Pre-Validation Study

**DOI:** 10.3390/jcm15010283

**Published:** 2025-12-30

**Authors:** Ewelina Trojacka, Joanna Przybek-Skrzypecka, Justyna Izdebska, Jacek P. Szaflik, Musa Aamir Qazi, Abdullah Azhar, Janusz Skrzypecki

**Affiliations:** 1 Center of Ocular Microsurgery, Professor Jerzy Szaflik’s Clinic in Warsaw, 00-215 Warszawa, Poland; 2 SPKSO Ophthalmic University Hospital in Warsaw, 03-709 Warszawa, Poland; 3 Department of Ophthalmology, Medical University of Warsaw, 02-091 Warszawa, Poland; 4 Department of Experimental Physiology and Pathophysiology, Medical University of Warsaw, 02-091 Warszawa, Poland

**Keywords:** visual symptoms questionnaire, PROM, cataract surgery, GenAI, LLMs-assisted PROM

## Abstract

**Background/Objectives:** Development of Patient-Reported Outcome Measures (PROMs) in ophthalmology is limited by high patient burden during early validation. We propose an In Silico Pre-validation Framework using Large Language Models (LLMs) to stress-test instruments before clinical deployment. **Methods:** The LLM generated a PROM questionnaire and a synthetic cohort of 500 distinct patient profiles via a Python-based pipeline. Profiles were instantiated as structured JSON objects with detailed attributes for demographics, lifestyle, and health background, including specific clinical parameters like IOL type (Monofocal, Multifocal, EDOF) and dysphotopsia severity. To eliminate memory bias, a stateless simulation approach was used for test–retest reliability; AI agents were re-instantiated without access to prior conversation history. Psychometric validation included Confirmatory Factor Analysis (CFA) using WLSMV estimation and Differential Item Functioning (DIF). **Results:** The model demonstrated excellent fit (CFI = 0.962, TLI = 0.951, RMSEA = 0.048, SRMR = 0.063), confirming structural validity. DIF analysis detected no significant bias based on age, sex, or IOL type (0/20 items flagged). Internal consistency was robust (Cronbach’s alpha > 0.80) and stateless test–retest reliability was high (ICC > 0.90), indicating stability independent of algorithmic memory. Convergent validity was established via significant correlations with NEI-VFQ-25 scores (Spearman’s: −0.425 to −0.652,). While responsive to change, known-groups validity reflected realistic clinical overlap. **Conclusions:** LLM-based pre-validation effectively mirrors complex human response patterns through “algorithmic fidelity.” By identifying structural failure points in silico, this framework ensures PROMs are robust and unbiased before clinical trials, reducing the ethical and logistical burden on real-world populations.

## 1. Introduction

Modern clinical practice is increasingly centered on improving the patient experience and overall quality of life, marking a paradigm shift from purely objective clinical metrics to subjective well-being [1,2,3,4,5]. A key component of this patient-centered approach is the use of patient-reported outcome measures (PROMs), which offer indispensable insights into patients’ subjective experiences, symptom burden and treatment satisfaction that cannot be captured by clinical examination alone [6,7,8,9,10,11,12,13,14]. However, the development of high-quality, scientifically robust PROMs is notoriously resource-intensive and time-consuming. It requires extensive input from diverse patient populations through iterative focus groups and cognitive debriefing. Often, inconsistencies, ambiguities, or critical omissions in the questionnaire items become apparent only after laborious rounds of patient interviews and statistical validation, leading to costly delays [15,16,17,18,19]. These logistical bottlenecks can significantly hinder the timely creation of sensitive and condition-specific tools, creating a measurement gap particularly in fast-evolving clinical fields where medical innovation outpaces instrument validation [15,16,17,20].

One such rapidly advancing field is cataract surgery with premium intraocular lenses (IOLs). Although advances in IOL technology—such as multifocal and extended depth of focus (EDOF) designs—have successfully reduced dependence on glasses, lenses that enhance pseudoaccommodation introduce a complex trade-off [21,22]. They may generate unwanted visual phenomena, such as glare, halos, starbursts, or reduced contrast sensitivity, which some patients find intolerable despite excellent visual acuity [23,24,25]. In rare but severe cases, these subjective symptoms may even prompt IOL explantation, highlighting a disconnect between objective surgical success and patient satisfaction [23,26,27,28,29]. Early detection and quantification of such adverse experiences are critical for both preoperative counseling and postoperative management. Yet, currently, only one validated PRO tool exists specifically for premium IOLs, while generic vision questionnaires often lack the granularity to detect subtle optical side effects [17]. There is a clear and urgent need for more tailored, design-specific PROMs that can support clinical trials and post-market surveillance of novel IOLs.

In parallel, the rapid advancement of large language models (LLMs) offers a transformative, yet underutilized, opportunity in medical research [30,31,32]. Although artificial general intelligence (AGI) remains a distant and evolving concept, current LLMs have demonstrated a remarkable capacity to simulate human-like language understanding and reasoning [33,34]. Beyond simple text generation, these models can be engineered to adopt distinct “personas”—simulating diverse demographic backgrounds, psychological profiles and clinical histories—to act as synthetic research subjects. This capability allows LLMs to mirror the multivariate distributions of human populations through a high degree of “algorithmic fidelity”. This enables the generation of synthetic patient responses that reflect nuanced symptoms, allowing for data-driven, scalable, and customizable approaches for PROM development that can potentially capture subtle descriptions often overlooked in routine clinical practice [35,36,37].

In this study, we present a systematic proof-of-concept framework for the generation and In Silico Pre-validation (Phase 0) of PRO questionnaires dedicated to cataract surgery using LLMs. By leveraging a Python-based automation pipeline, this approach demonstrates how instruments can be tailored to specific IOL designs with unprecedented speed and efficiency. Our methodology utilizes a synthetic cohort of 500 patients instantiated as structured JSON objects to identify relevant symptom domains. To rigorously evaluate psychometric stability, we employ a stateless simulation design—where AI agents are re-instantiated for reliability testing without access to prior conversation history—to eliminate memory bias. While this pre-validation approach is not intended to replace human clinical trials, it offers a transformative potential to accelerate the traditionally slow pipeline of PROM development. By providing a scalable environment to “stress-test” instrument structures using Confirmatory Factor Analysis (CFA) and Differential Item Functioning (DIF) before clinical deployment, this framework ensures that the methodological foundation of patient-reported tools can keep pace with rapid surgical innovation.

## 2. Materials and Methods

### 2.1. Study Design and AI Framework

During the preparation of this study, the author(s) used Chat GPT-4o for the purposes of data collection, analysis and interpretation of data. The authors have reviewed and edited the output, and take full responsibility for the content of this publication.

This study utilized a Generative Artificial Intelligence framework to develop and psychometrically validate a new Patient-Reported Outcome Measure (PROM) for patients undergoing cataract surgery with various Intraocular Lens (IOL) implants, including premium (e.g., multifocal, extended depth of focus [EDOF]) and monofocal designs. The instrument development and validation process was conducted in silico using Chat GPT-4o (OpenAI, San Francisco, CA, USA) following the iterative development process recommended by the U.S. Food and Drug Administration (FDA) guidance on PRO measures [15].

### 2.2. Instrument Development

The 20-item questionnaire was developed through a multi-stage prompt engineering process designed to establish content validity. First, the LLM simulated focus groups with synthetic patient personas to identify the concept of interest, visual quality and daily functioning after IOL implantation. Prompts were designed to elicit open-ended feedback until saturation was achieved, ensuring all relevant symptoms were captured.

To minimize redundancy and ensure broad conceptual coverage, the selection of candidate items was refined using a Maximal Marginal Relevance (MMR) algorithm based on sentence embeddings (SentenceTransformers). This algorithmic approach utilized cosine similarity to mathematically optimize the trade-off between semantic diversity and relevance to the construct, ensuring that selected items covered distinct aspects of the patient experience rather than repeating similar concepts.

Based on these qualitative insights, the LLM generated a 20-item instrument utilizing a 5-point Likert scale to assess symptom frequency and severity. The items cover five distinct domains: Near/Reading, Intermediate/Screen & Focus, Distance/Night & Dysphotopsia, Symptoms/Asthenopia & Lighting, and Daily Function/Independence.

**Figure 1:**
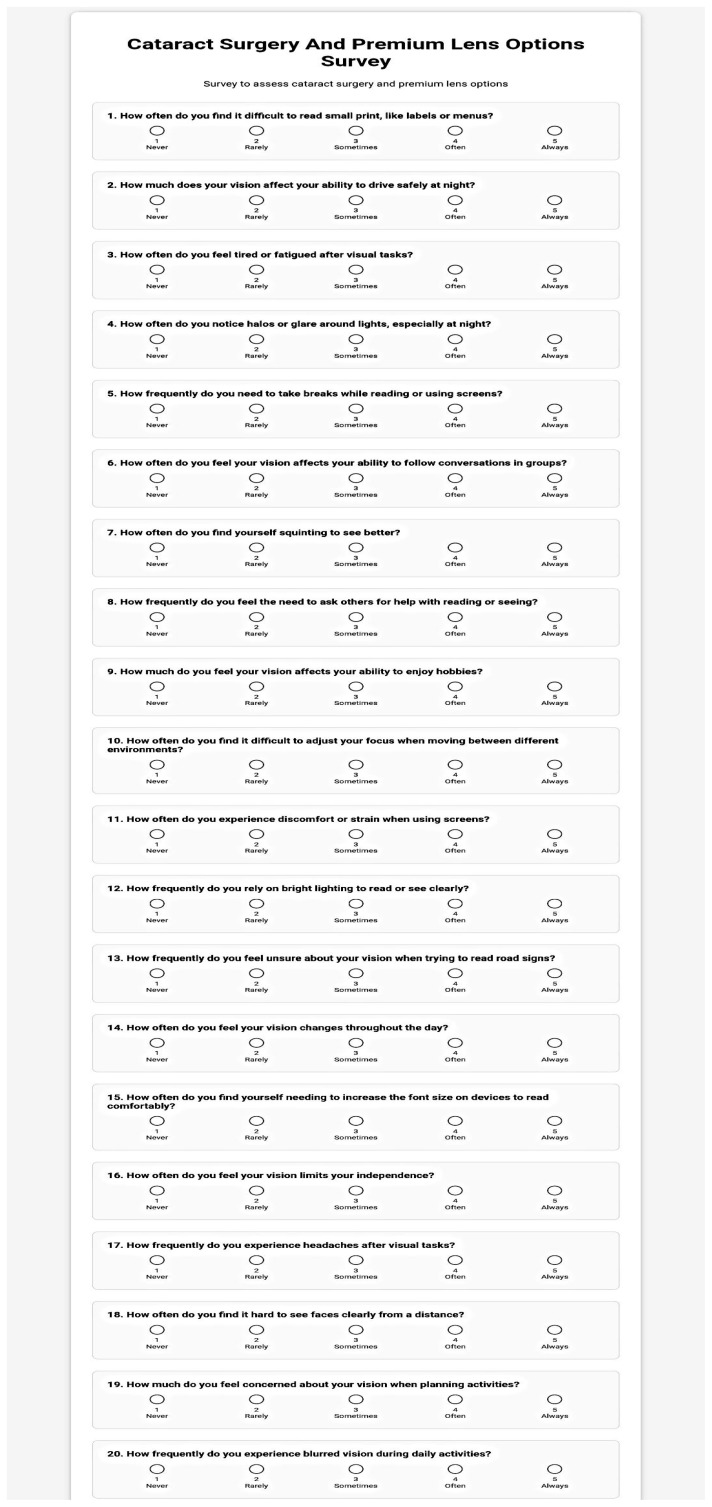
20-item questionnaire generated with the assistance of LLMs.

Figure 1 provides an overview of the developed 20-item questionnaire.

To ensure clarity and relevance, simulated cognitive interviewing was performed to refine item wording prior to validation.

### 2.3. Synthetic Population and Data Generation

To validate the instrument, the LLM generated a synthetic cohort of 500 distinct patient profiles representing the intended target population of cataract surgery patients. This process was automated using a Python-based pipeline (Python 3.8+, Beaverton, OR, USA) where patient profiles were instantiated as structured JSON objects containing detailed attributes for demographics, lifestyle, health background and psychological profile. These profiles were characterized by specific clinical parameters (IOL type [Monofocal, Multifocal, EDOF], laterality of surgery, time since surgery, dysphotopsia severity and spectacle independence). The model was instructed to adopt these specific patient personas to generate survey responses.

Consistent with reliability testing protocols, a test–retest design was employed where the LLM generated paired observations for the 500 synthetic patients with a simulated 1-week interval. To technically enforce this design and eliminate memory bias, a stateless simulation approach was utilized. For the retest time point, AI agents were re-instantiated using the identical JSON persona profiles but without access to their prior conversation history. The simulated time lapse was introduced solely through contextual system prompts, treating each survey completion as an independent probabilistic event conditioned only on the persona’s fixed identity and the specific time-point context. This method ensured stability while preventing the “testing effect” often observed in human subjects.

### 2.4. Psychometric and Statistical Analysis

Psychometric validation followed established guidelines. Data analysis was performed using Python 3.8+ (libraries: pandas, numpy, scipy, sklearn).

### 2.5. Scoring

Domain scores were calculated as the mean of available items, requiring ≥ 70% completion. A Global Symptom Burden score was derived from the mean of the available domain scores.

### 2.6. Reliability

Internal consistency was assessed using Cronbach’s alpha with 95% confidence intervals (target: alpha ≥ 0.80). Test–retest reliability was evaluated using the Intraclass Correlation Coefficient (ICC 2.1) for absolute agreement (target: ICC ≥ 0.75) to demonstrate score stability. Measurement error was quantified using the Standard Error of Measurement (SEM) and Minimal Detectable Change at 95% confidence (MDC95).

### 2.7. Validity

Structural validity was assessed via Confirmatory Factor Analysis (CFA) using WLSMV estimation suitable for ordinal data to confirm the conceptual framework. Model fit was considered acceptable if the Comparative Fit Index (CFI) and Tucker–Lewis Index (TLI) ≥ 0.95, Root Mean Square Error of Approximation (RMSEA) ≤ 0.06, and Standardized Root Mean Square Residual (SRMR) ≤ 0.08. Known-groups validity was tested using Kruskal–Wallis tests to compare scores across clinical strata (e.g., IOL type).

### 2.8. Fairness and Responsiveness

Differential Item Functioning (DIF) was analyzed using ordinal logistic regression stratified by age, sex and IOL type to ensure the instrument is unbiased across subgroups. Ability to detect change (responsiveness) was evaluated using Cohen’s d, standardized response mean and Guyatt’s responsiveness index. Minimal Important Difference (MID) estimates were calculated combining distribution-based (0.5 SD, SEM) and anchor-based (PGRC) approaches using ROC analysis to define meaningful within-person change.

## 3. Results

Structural Validity and Fairness CFA was conducted to verify the hypothesized five-domain structure of the instrument. The model demonstrated excellent fit to the data, satisfying all pre-specified criteria (CFI = 0.962, TLI = 0.951, RMSEA = 0.048, SRMR = 0.063), confirming the structural validity of the PROM (Table 1).

Furthermore, DIF analysis confirmed the fairness of the instrument across key demographic and clinical subgroups. No items displayed significant bias based on age, sex, or IOL type (Table 1).

Item Characteristics and Reliability Descriptive analysis of the 20 items revealed high data quality with missing data rates below 3% and negligible ceiling effects (<1%). The instrument demonstrated robust reliability across all domains (Table 2).

Internal consistency was excellent (Cronbach’s alpha > 0.80) and test–retest reliability over a 1-week interval was high (ICC > 0.90), indicating score stability. Measurement error was quantified using SEM and MDC95.

### 3.1. Construct Validity

Convergent validity was established through significant correlations with the NEI-VFQ-25 Composite score. As anticipated, all domains showed moderate-to-strong negative correlations (ranging from −0.425 to −0.652), indicating that higher symptom burden is associated with lower vision-related quality of life (Table 2). Known-groups validity was assessed by comparing scores across clinical strata; however, in this synthetic dataset, differences between groups did not reach statistical significance (*p* > 0.05).

### 3.2. Responsiveness and Minimal Important Difference

The instrument was responsive to change, with significant effect sizes detected in most domains. MID estimates were derived to aid clinical interpretation; anchor-based MIDs are presented in Table 2, while distribution-based estimates (0.5 SD) yielded consistent ranges (0.29–0.44).

## 4. Discussion

This study presents a novel In Silico Pre-validation Framework (Phase 0) for the development and preliminary simulation of a PROM targeting the visual symptomatology associated with premium and monofocal IOL implantation. Leveraging the generative capabilities of LLMs through a Python 3.8+-based automation pipeline, we successfully synthesized a 20-item instrument that demonstrates robust structural consistency within a simulated environment. These findings offer initial evidence for theoretical frameworks proposing that LLM-assisted development can achieve high methodological rigor in the instrument-design phase while substantially optimizing resource allocation [35]. However, this study underscores that such in silico results serve as a foundational step, requiring subsequent clinical validation to account for the full spectrum of human sensory experience. By simulating a diverse cohort of 500 patient profiles, instantiated as structured JSON objects and characterized by specific demographic and clinical parameters, we demonstrated that generative AI can effectively emulate the iterative item-generation and refinement phases traditionally conducted through labor-intensive qualitative research.

Traditional PROM development, exemplified by the 37-item Assessment of IntraOcular Lens Implant Symptoms (AIOLIS), necessitates extensive longitudinal investment, involving patient recruitment, manual qualitative coding, and iterative expert review [17]. While existing tools like AIOLIS provide granular symptom assessment, their static nature limits rapid adaptation to evolving IOL technologies [17]. In contrast, the framework utilized in this study facilitated the rapid simulation of heterogeneous patient personas to generate a concise, FDA-aligned instrument. The resulting 20-item questionnaire maintains content breadth across five distinct functional domains—Near, Intermediate, Distance, Symptoms, and Function—while reducing respondent burden compared to legacy instruments.

A critical finding of this study is that the acceleration of the development timeline did not compromise psychometric integrity. The instrument exhibited excellent internal consistency across all domains, with Cronbach’s alpha coefficients ranging from 0.807 to 0.934, and demonstrated high temporal stability. To address concerns regarding “algorithmic memory” or repetitive logic, we employed a stateless simulation approach where AI agents were re-instantiated for reliability testing without access to prior conversation history. This method yielded test–retest ICCs exceeding 0.90, confirming that score stability is a property of the instrument’s conceptual clarity rather than AI memory bias. Furthermore, Confirmatory Factor Analysis (CFA) using WLSMV estimation provided strong evidence for structural validity. The model fit indices (CFI = 0.962, TLI = 0.951, RMSEA = 0.048, SRMR = 0.063) satisfied stringent criteria, confirming that the five-factor structure meaningfully represents the latent constructs of visual quality in the target population. Additionally, the absence of Differential Item Functioning (DIF) across age, sex, and IOL type (0/20 items flagged) suggests the instrument is robust against measurement bias, satisfying the regulatory requirement for fairness in PROM design.

The instrument demonstrated statistically significant responsiveness to change, with Minimal Important Difference (MID) estimates established via both anchor-based and distribution-based methods. While the observed effect sizes were modest (Cohen’s), these values warrant careful interpretation within the specific context of ophthalmic PROMs where small numerical shifts frequently correspond to the MID. Convergent validity was corroborated by significant negative correlations with the NEI-VFQ-25 composite score (Spearman’s from −0.425 to −0.652), aligning with established quality-of-life metrics. Notably, known-groups validity analyses did not yield statistically significant differentiations between strata of dysphotopsia severity. This lack of differentiation is likely attributable to the inherent challenges of simulating the full spectrum of clinical variability via synthetic cohorts. Consequently, future iterations of this framework should employ targeted “stress-testing” through the oversampling of extreme synthetic personas to ensure the instrument maintains robust discriminative power across the most severe clinical scenarios.

### 4.1. Future Directions and Clinical Applications

The implications of this proof-of-concept framework extend far beyond the development of a single instrument. The underlying architecture explored in this study—specifically the generation of structured patient personas and the semantic ranking of diagnostic items—lays the groundwork for integrating “Digital Twins” into preoperative counseling [38,39,40,41]. In the future, by inputting real-world parameters such as biometry, lifestyle preferences, and personality traits into such models, clinicians could generate a patient-specific digital surrogate. Simulating postoperative scenarios on these surrogates could help surgeons anticipate subjective complaints before surgery.

Furthermore, the stateless simulation method utilized here provides a foundation for the development of Dynamic Conversational PROMs [34,37,42,43,44,45,46,47]. Unlike static paper-based questionnaires, future LLM-driven tools could function as “active listeners”. By leveraging the semantic similarity logic explored in our framework, these systems could provide AI-driven Computerized Adaptive Testing (CAT), prioritizing the most relevant questions based on real-time responses to drastically reduce patient burden.

### 4.2. Limitations

This study must be interpreted within the context of its in silico design. While the LLM successfully generated a synthetic cohort of 500 patient profiles, the generated responses may lack the stochastic variability inherent to human subjects [48]. Consequently, while the structure and reliability of the tool are well-supported, the magnitude of clinical responsiveness requires verification in real-world populations.

## 5. Conclusions

In conclusion, this study demonstrates that LLM-assisted methodologies represent a paradigm shift in psychometrics, enabling the efficient generation of structurally sound and reliable PROMs. The developed Visual Symptoms Questionnaire satisfies core psychometric requirements for reliability, validity, and fairness. It offers a scalable, adaptable tool for assessing visual outcomes in the modern era of refractive cataract surgery, bridging the gap between rapid technological innovation in IOL design and the need for rigorous patient-centered assessment.

## Figures and Tables

**Table 1:** Structural Validity (CFA) and Fairness Assessment.

Metric Category	Statistic/Analysis	Value/Result	Criterion/Interpretation	Status
Structural Validity (CFA *)	CFI (Comparative Fit Index)	0.962	≥0.95 (Good fit)	Met
	TLI (Tucker–Lewis Index)	0.951	≥0.95 (Good fit)	Met
	RMSEA (Root Mean Sq. Error)	0.048	≤0.06 (Good fit)	Met
	SRMR (Std. Root Mean Sq. Residual)	0.063	≤0.08 (Good fit)	Met
Fairness (DIF *)	Items Flagged	0/20	*p* < 0.05 (adj) & ΔR^2^ ≥ 0.02	Passed
	Variables Tested	Age, Sex, IOL Type	No significant bias detected	Passed

* CFA = Confirmatory Factor Analysis; DIF = Differential Item Functioning.

**Table 2:** Domain-Level Psychometric Properties: Reliability, Validity and Responsiveness.

Domain	Internal Consistency (Cronbach’s α)	Test–Retest Reliability (ICC [95% CI])	Measurement Error (SEM/MDC95)	Convergent Validity (Spearman’s ρ)	Responsiveness (Cohen’s d)	MID Estimate (Anchor- Based)
Near/Reading	0.807	0.915 [0.898, 0.928]	0.22/0.62	−0.425 *	0.102	1.00
Intermediate/ Screen & Focus	0.897	0.951 [0.941, 0.959]	0.19/0.52	−0.551 *	0.140	0.33
Distance/Night & Dysphotopsia	0.934	0.960 [0.953, 0.967]	0.17/0.48	−0.603 *	0.140	0.17
Symptoms/ Asthenopia	0.925	0.964 [0.957, 0.970]	0.15/0.43	−0.652 *	0.144	0.20
Daily Function/ Independence	0.885	0.965 [0.959, 0.971]	0.11/0.31	−0.589 *	0.061	0.13

* *p* < 0.001. SEM = Standard Error of Measurement; MDC95 = Minimal Detectable Change at 95% confidence; MID = Minimal Important Difference; ICC = Intraclass Correlation Coefficient.

## Data Availability

Dataset available on request from the authors.
